# Using Actigraphy to Measure Sleep Patterns in Rheumatoid Arthritis: A Pilot Study in Patients Taking Night-Time Prednisone

**DOI:** 10.1002/msc.1052

**Published:** 2013-05-20

**Authors:** Lynsey L Clarke, Sue Wilson, John R Kirwan

**Affiliations:** 1 University of Bristol Academic Rheumatology Unit, Bristol Royal InfirmaryBristol, UK; 2 Academic Unit of Psychiatry, School of Social and Community Medicine, University of BristolBristol, UK

**Keywords:** Rheumatoid arthritis, sleep, glucocorticoids, actigraphy

## Abstract

**Objective:**

Poor sleep quality is a commonly reported but under-investigated consequence of rheumatoid arthritis (RA). Actigraphy is a non-invasive way of measuring sleep, estimated from the frequency and intensity of physical movement at the wrist. We used actigraphy to measure sleep parameters compared with sleep questionnaire data, and assessed the practicality of actigraph use in patients with RA.

**Methods:**

In a pilot study of actigraphy conducted within an investigation of night-time prednisone treatment and circadian interleukin-6 concentrations in ten patients with active RA, we compared actigraphy with the St Mary’s Hospital Sleep Questionnaire and assessed whether night-time administration of prednisone resulted in increased sleep disturbance.

**Results:**

The actigraph watch was well tolerated by our patients, producing adequate data for analysis for 128 out of 133 test days (96.2%). The results indicated reasonable concordance between actigraph and sleep questionnaire data in the present sample. Patient satisfaction with sleep (question 11) strongly correlated with sleep efficiency measured by the actigraph (*r* = 0.71, *p* = 0.22) and showed a trend for inverse correlation with the fragmentation index (*r* = −0.60, *p* = 0.067). Quality of sleep (question 9) correlated non-significantly with the fragmentation index (*r* = −0.59, *p* = 0.072). We were unable to identify any significant correlations between clinical measures of disease and sleep parameters in this sample. There were no apparent detrimental consequences of the night-time dose of prednisone on the measures of sleep quality and quantity.

**Conclusion:**

In spite of the physical disability imposed by RA, the actigraph was well tolerated and gave a useful measure of sleep in patients with active disease. It has the potential for use in larger controlled trials.

## Background

**Figure 1:**
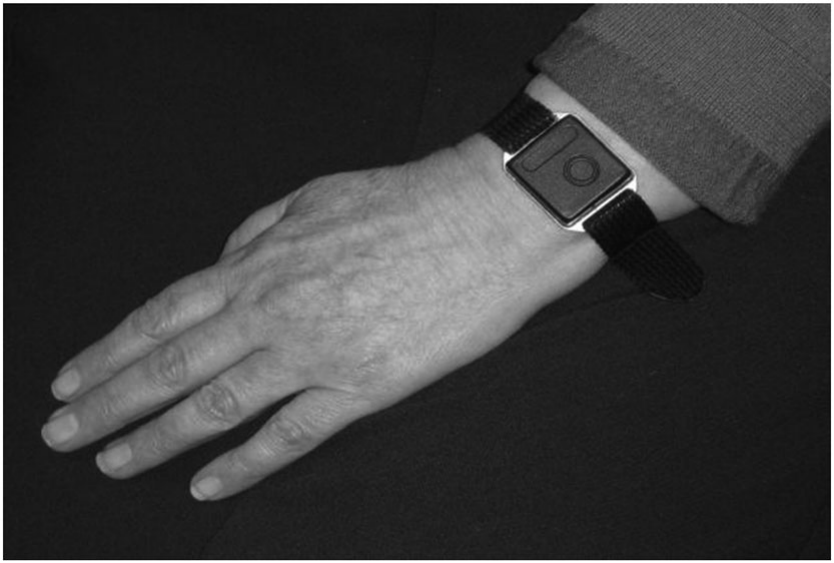
The actigraph in use

Although sleep disturbance has not been studied widely in rheumatoid arthritis (RA), it has been identified by patients as an important consequence of their disease that merits further investigation (Kirwan et al., 2003; Wells et al., 2009). It is commonly reported, by up to 50% of patients (Drewes et al., 1998; Nicassio and Wallston, 1992). Subjective reports by RA patients have been found to overestimate sleep efficiency (SE) (Hirsch et al., 1994; Mahowald et al., 1989), highlighting the need for objective measures of sleep quality and quantity in addition to patient-reported outcomes. The gold standard for measuring sleep is polysomnography, which involves several measurements being collected simultaneously, such as electroencephalography (EEG), eye movements and lower limb electromyography (EMG). It is usually performed in a sleep laboratory and is therefore inconvenient for the subject, expensive, time consuming and takes the subject away from their normal sleeping environment; it is rarely possible to use over more than two or three consecutive nights. Further, studies using polysomnography have provided conflicting results comparing SE in RA patients and controls (Drewes et al., 1998; Hirsch et al., 1994). These limitations mean that polysomnography can be impractical as a tool in clinical trials. By contrast, an actigraph is an accelerometer that is worn on the wrist like a watch (Ancoli-Israel et al., 2003) (Figure 1) and measures the frequency and intensity of physical activity. It is simple, non-invasive, inexpensive and convenient, and can be used in the patient’s normal environment over the course of days or weeks. Small studies using actigraphy have suggested that RA patients may have poor sleep quality (Lavie et al., 1992), and further work is needed to investigate its utility in larger trials in RA.

The possible causes of sleep disturbance in RA are not clear but may be related to several factors, including disease symptoms such as pain (Drewes et al., 2000; Lee et al., 2009; Nicassio and Wallston, 1992), stress (Treharne et al., 2007) and fatigue (Goodchild et al., 2010) or sleep disorders such as obstructive sleep apnoea (Gjevre et al., 2012). Medication may contribute, as exogenous glucocorticoid administration may alter normal sleep patterns (Gillin et al., 1972; Vgontzas and Chrousos, 2002). In addition, circulating inflammatory mediators such as interleukin-6 (IL-6) may also be involved, as studies in healthy controls have shown that higher concentrations are associated with fatigue, insomnia and sleep deprivation (Mastorakos et al., 1993; Vgontzas and Chrousos, 2002; Vgontzas et al., 1999, 2002). Recent reports confirm that serum IL-6 is substantially raised in patients with RA and that it increases during the night, reaching a maximum concentration at about 8 a.m. (Clarke et al., 2011; Perry et al., 2010).

Night-time glucocorticoid administration can substantially suppress the raised night-time IL-6 concentrations in patients with RA, although not necessarily achieve a completely normal concentration (Clarke et al., 2011). Clinically, patients achieve a noticeable improvement in morning stiffness compared with daytime glucocorticoid administration (Buttgereit et al., 2008) but the outcome in relation to sleep quality and duration is not known. Therefore, as a preliminary approach to evaluating the acceptability of actigraphs for prolonged use and making an initial assessment of the possible effects of medication and disease control on sleep patterns, we took the opportunity to measure sleep using subjective patient reports and actigraph measurements as part of a study looking at the effects on circadian variation in cytokines of a low dose of prednisone timed to be released at 2 a.m. (Clarke et al., 2011). Our aims were:

1. To evaluate the acceptability and practicality of using the actigraph in these patients;
2. To measure physical activity during the night as a surrogate marker of sleep quality and quantity, and assess the findings in relation to a widely used sleep questionnaire;
3. To make a preliminary evaluation of whether night-time glucocorticoid administration caused any obvious or substantial change in sleep pattern.

## Materials and methods

### Patients and assessments

The study was approved by the Central and South Bristol Research Ethics Committee UK and was registered with Controlled-Trials.com: ISRCTN 17552423. Ten patients (seven female), aged 51–79 years {mean [standard deviation (SD)] 64.7 (10.0) years}, with active RA (Clarke et al., 2011), recruited within the University of Bristol Academic Rheumatology Unit at Bristol Royal Infirmary, gave informed written consent. Active disease was defined by greater than three tender and swollen joints, pain of at least 30 mm as measured on a 100 mm visual analogue scale (VAS; anchors were ‘no pain’ to ‘severe pain’), a C-reactive protein (CRP) of at least 15 mg/L or an erythrocyte sedimentation rate (ESR) of at least 30 mm/hour. Patients had a mean (SD) disease duration of 17.0 (10.1) years, all had erosions on X-rays of the hands and feet, and all were on stable medical therapy. Patients had received no glucocorticoids by any route in the preceding three months and had never been treated with tumour necrosis factor antagonists or other biological therapies. Treatment with disease-modifying drugs and other medications had been stable for at least three months. On the first study day, patients completed the St Mary’s Hospital Sleep Questionnaire (SMHSQ) (Ellis et al., 1981), a widely used instrument which asks patients to report on different aspects of sleep from the previous night, and underwent a clinical assessment including standardized 28-swollen and tender joint counts (Prevoo et al., 1995), pain in the last 24 hours (100 mm VAS labelled ‘no pain’ and ‘severe pain’), average duration of early morning joint stiffness over the previous three days in minutes (EMS), patient global assessment (100 mm VAS answering ‘Considering all the ways your arthritis affects you, please mark on the line how well you think you are doing’, with anchors ‘very well’ and very badly’), physician’s global assessment (100 mm VAS answering ’Clinician’s opinion of disease’, with anchors ‘none’ and ‘severe’), self-reported disability (Health Assessment Questionnaire; HAQ) (Fries et al., 1980) and Disease Activity Score (DAS28) (Prevoo et al., 1995).

Patients wore an actigraph watch (Actiwatch, Cambridge Neurotechnology Ltd, Cambridge, UK) on the non-dominant wrist continuously throughout the following two weeks of the study and removed it only for bathing and swimming. Patients underwent a 24-hour blood sampling regimen (Clarke et al., 2011) and then took 5 mg timed-release prednisone tablet medication each evening at 10 pm for two weeks. The prednisone has a coating designed to release the active drug approximately four hours post-ingestion to target the highest overnight plasma cytokine concentrations (Buttgereit et al., 2008). Patients returned to complete the sleep questionnaire, undergo a further clinical assessment and have a further 24-hour blood sampling. We noted patients’ comments on the ease or otherwise of wearing the actigraph watch, whether they had removed it at any time and whether it would be acceptable for longer-term use.

The actigraph data (the activity counts measure any activity sufficient to displace the internal transducer in the actigraph, expressed as mean activity over one-minute intervals) were downloaded to a computer which used associated software (Actiwatch Activity and Sleep Analysis, Cambridge Neurotechnology Ltd) to generate a display of the data for each day that the actigraph had been worn and applied a validated algorithm (Meadows et al., 2005) to estimate sleep parameters. These included SE (percentage of time in bed actually spent asleep), actual sleep time (AST) and fragmentation index [FI; sum of the movement time (%) and the number of movement episodes during sleep periods] for each 24-hour period. Actigraph data only from nights spent in the usual environment were included, excluding the 24-hour periods at the beginning and end of the study, when the patients were resident in an overnight research facility for regular blood sampling. SE is calculated by the actigraph software based on overnight activity. AST is estimated from the duration of low-level or absence of activity counts. The FI is a measure of restlessness based on the frequency and amplitude of movements overnight. The higher the FI, the more restless the patient has been. Although the total length of the study was slightly different for different patients, all patients had at least 12 consecutive home sleep nights measured. The sample size of ten patients was determined by the overnight study requirements, but we would have expected clinically meaningful differences to be apparent, based on previous experience. The mean, SD and 95% confidence intervals (CIs) were calculated for nights 1–6 (week 1 of prednisone treatment) and nights 7–12 (week 2 of prednisone treatment). Activity counts and patterns over the two-week period were assessed for changes during the prednisone treatment by visual assessment, correlation coefficients of the mean values over the two weeks and paired *t*-tests of mean values for week 1 compared with week 2. We also sought correlations (Pearson correlation coefficient) between the actigraph data and the answers to the relevant sleep questionnaire items (questions 6, 7, 9 and 11) at the end of the study, and clinical assessments.

## Results

**Figure 2:**
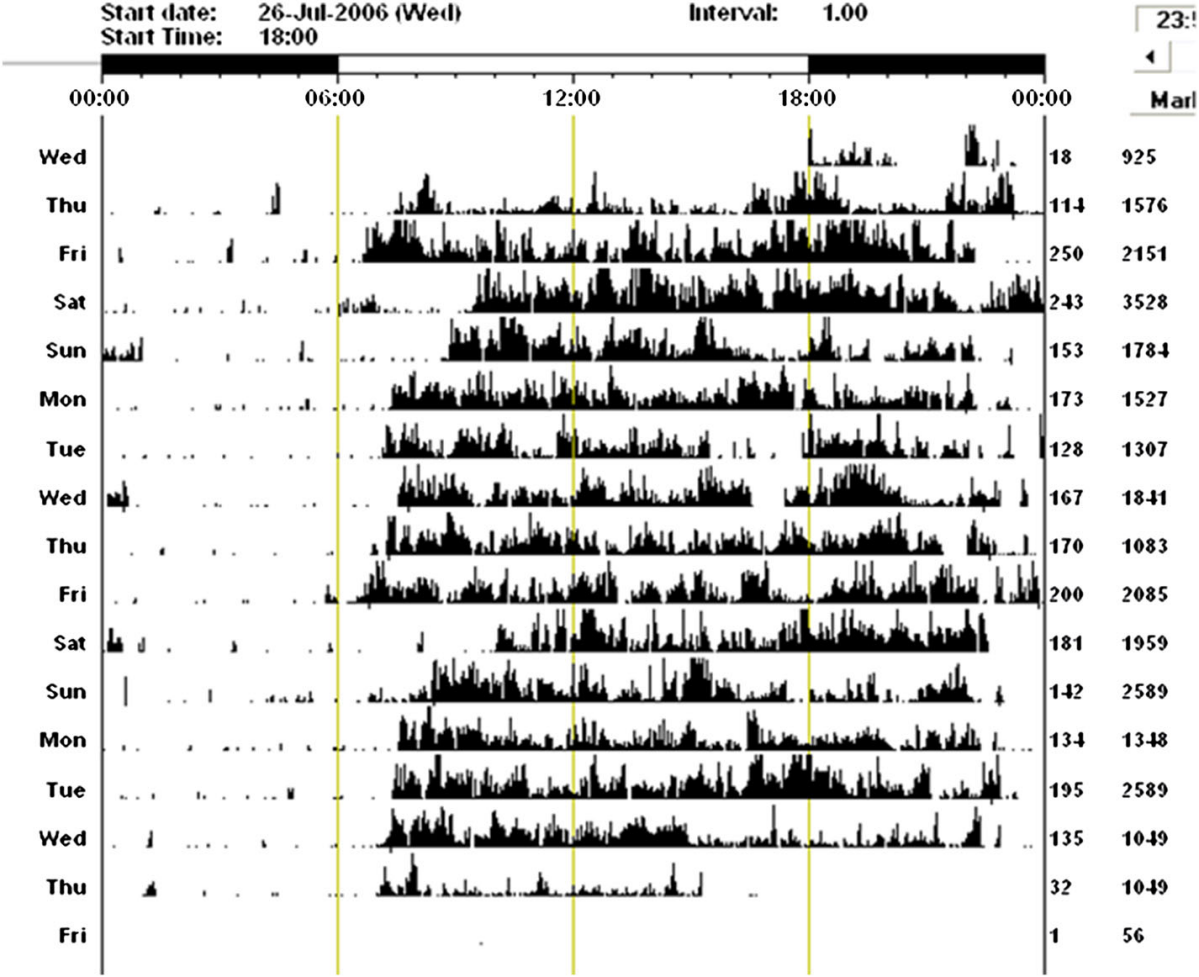
A typical actigraph readout for one patient

All patients were able to tolerate the actigraph well and were happy to recommend further use of actigraphs. Adequate data for analysis were available for 128 out of 133 test days (96.2%). Occasionally, the actigraph was removed or malfunctioned, resulting in inadequate data for three days spread across the two weeks in one patient and for one day in each of two other patients. A typical actigraph output for one patient is shown in Figure 2; a clear separation between non-movement during sleep and frequent movement during waking hours can be seen. From time to time during the night, there were small bursts of movement, indicating disturbances in sleep.

**Figure 3:**
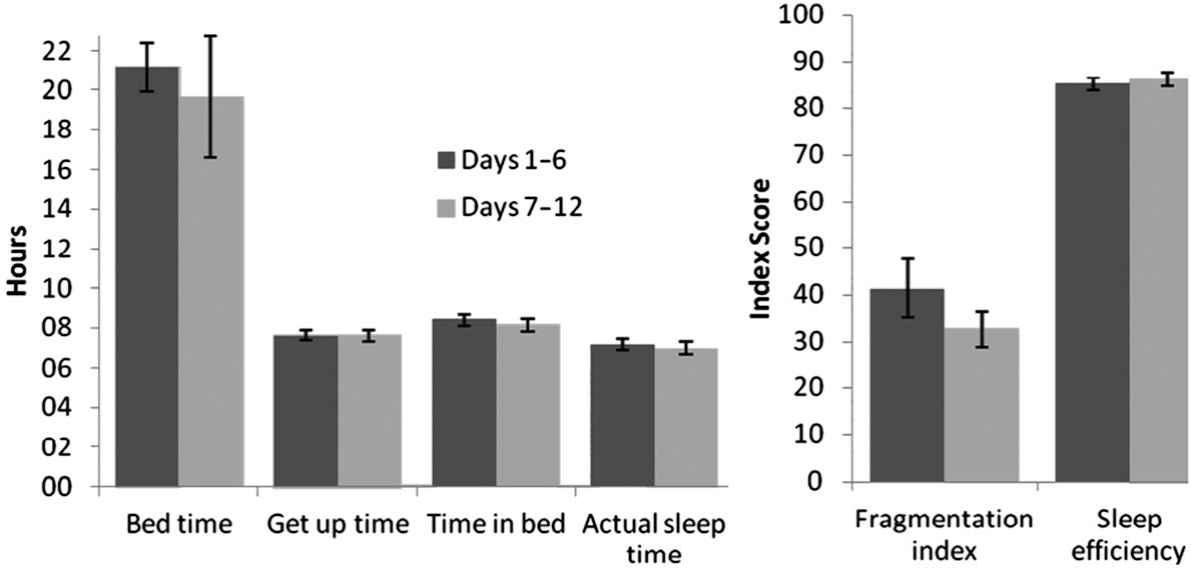
Mean (95% confidence interval) for actigraph measurements and indices for week 1 compared with week 2

**Figure 4:**
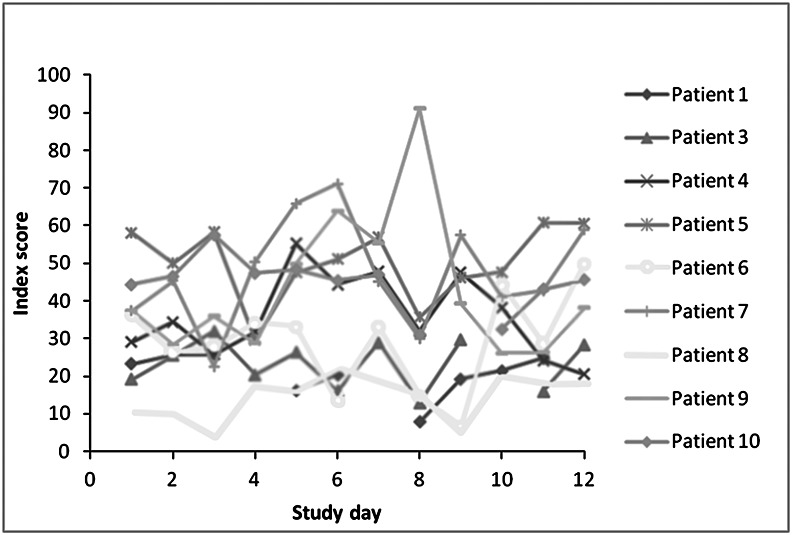
Fragmentation index for each patient for each study day (patient 2 outlier excluded)

Figure 3 shows the changes in mean sleep time, SE and FI scores during each of the two weeks of measurement. There were no significant differences in the scores taken together from week 1 compared with those from week 2, but the lower score for FI in week 2 had a significance of *p* = 0.072 on a paired *t*-test. However, there were large variations in actual scores between patients and within patients from day to day (Figure 4), and one patient (patient 2) showed an unusually high FI score for week 1. On investigation, this was most likely due to an actigraph malfunction affecting FI only. If patient 2 is omitted, there is no suggestion of a difference in mean FI values between weeks 1 and 2 for the patients taken together [mean (95% CI) 34.7 (2.8) versus 34.7 (3.9); *p* = 0.91). AST showed only a minor, non-significant difference between the first week (7 hours 15 minutes) and second week (7 hours 5 minutes’ difference 10 minutes; *p* > 0.1).

We compared the three measures of sleep quality with the equivalent questions in the SMHSQ (Table 1). With only ten patients, a correlation coefficient would have to be > 0.631 for statistical significance at *p* < 0.05. Nevertheless, patient satisfaction with sleep (question 11) correlated strongly with sleep efficiency measured by the actigraph (*r* = 0.71, *p* = 0.022) and showed a trend for an inverse correlation with FI ( *r*= −0.60, *p* = 0.067). Quality of sleep (question 9) correlated non-significantly with FI (*r* = −0.59, *p* = 0.072). Although the remainder of these coefficients were not statistically significant, the pattern of the relationships was appropriate. None of the self-report questions correlated with measured AST.

**Table 1.** Correlations between the St Mary’s Hospital Sleep Questionnaire (SMHSQ) questions and actigraph measures

SMHSQ questions	Fragmentation index	Sleep efficiency	Actual sleep time
Q6 No. of awakenings?	0.47	−0.29	−0.14
Q7 How much sleep (minutes)?			−0.18
Q9 How well slept?	−0.59	0.45	−0.10
Q11 How satisfied with sleep?	−0.60	0.71*	0.30

* *p* < 0.05

Clinical measures of disease activity improved over the two weeks of observation: mean (95% CI) reduction in EMS 155 minutes (65–243); pain 18.6 (5.8–31.4); patient global assessment 19.6 (9.6–29.6); CRP 11.2 (1.5–20.9); DAS 0.67 (0.23–1.10). However, there was no correlation between these measures at the start and the actigraph sleep indices for week 1, or between the clinical measures at the end and actigraph indices in week 2.

Finally, we asked whether sleep quality and quantity changed over the two-week period while patients were taking night-time modified-release prednisone by comparing the pre- and post-treatment sleep questionnaire; this showed no significant change (Table 2).

**Table 2.** Comparison of St Mary’s Hospital Sleep Questionnaire (SMHSQ) responses before and after treatment

	Before treatment			After treatment				
SMHSQ questions	Mean	95% CI	Mean	95% CI
Q6 No. of awakenings?	2.50	(1.20–3.80)	2.10	(1.30–2.90)
Q7 How much sleep (min)?	376	(326–426)	397	(297–497)
Q9 How well slept?	3.80	(2.00–5.60)	4.30	(3.40–5.20)
Q11 How satisfied with sleep?	3.00	(1.40–4.60)	2.90	(1.50–4.30)

CI, confidence interval.

## Discussion

In response to the expressed wishes of patients with RA and experts in the field of outcome measurement in rheumatology (Kirwan et al., 2003), we addressed the issue of measuring sleep quality and quantity in RA as a pilot test of practicality by adding a sleep measure to an already planned clinical study. This was therefore an initial exploration of the use of the actigraph, and had modest aims. Although the number of patients was small (*N* = 10), all were able to tolerate the actigraph recorder, with only one patient having difficulties on three out of 15 days and most patients reporting no problems at all. Visual inspection of the actigraph recordings shows that there were clear differences between night-time and daytime movement patterns, and the calculated actigraph indices SE and FI were in line with the equivalent patient self-reported outcomes in the SMHSQ. We found no clear correlation between reported and measured aspects of sleep and measures of disease activity, but patients in the present study were chosen because they had high levels of disease activity. We found no evidence of deterioration in sleep during the time of the study, when patients were taking modified-release tablets of prednisone at night.

There are several limitations which prevent a wider interpretation of our detailed findings, the main ones being the small number of patients, the relatively short study period and the lack of pre-treatment actigraph measurements. In addition, the patients were not blinded to treatment (as this was primarily a biochemical study) and there were no controls. In spite of these limitations, however, it seems reasonable to conclude that the actigraph can be used on a much larger scale, and as part of a full randomized, controlled trial in which change in sleep is a primary or secondary outcome measure. Overall, the actigraph and the sleep questionnaire show reasonable correlation in measures of sleep quality but not sleep quantity. This may mean that RA patients may be good at estimating how well they have slept but not how long, a result which differs from previous research findings (Lavie et al., 1992). Wilson et al. (1998) have demonstrated the importance of multi-method assessment in patients with chronic musculoskeletal pain due to poor correlations between objective and subjective measures.

Although it seems possible that taking glucocorticoid medication at night might result in poor sleep with increased wakefulness, our results suggest that prednisone at a dose of 5 mg released at 2 a.m. does not significantly impair sleep, even when taken at night. A more robust assessment of this should be undertaken before a final conclusion can be drawn, and using the actigraph appears to be a practical approach to doing this, but it seems likely that a substantial deterioration in sleep quality is ruled out by our results. However, in future studies, analysis of circadian parameters using non-parametric methods, as used in other patient studies, may reveal subtle changes in the daily sleep–wake routine (Hatfield et al., 2004).

Although we did not find clinical measures to be associated with poor sleep in these patients, as noted above, the inclusion criteria for the study were designed for other purposes and recruited only patients with currently active joint disease. It would be interesting to assess patients with a range of disease activity, to look for correlations with sleep parameters. Hirsch et al. (1994) found no such correlation within 19 RA patients but, again, these patients were selected because they had active disease. There are many other variables that may influence sleep in RA patients, such as age, body mass index, depression and anxiety. Larger numbers of patients, particularly in the setting of a clinical trial, would allow further investigation of these and the role of disease activity and treatments such as prednisolone or cytokines such as IL-6 on sleep patterns in RA patients. A combination of self-report questionnaire and wearing the actigraph offers a practical methodology.
